# Nitrogen:phosphorous supply ratio and allometry in five alpine plant species

**DOI:** 10.1002/ece3.2587

**Published:** 2016-11-22

**Authors:** Xi Luo, Susan J. Mazer, Hui Guo, Nan Zhang, Jacob Weiner, Shuijin Hu

**Affiliations:** ^1^ College of Resources and Environmental SciencesNanjing Agricultural UniversityNanjingJiangsuChina; ^2^ Department of EcologyEvolution & Marine Biology, University of California, Santa BarbaraSanta BarbaraCAUSA; ^3^ Department of Plant and Environmental SciencesUniversity of CopenhagenFrederiksbergDenmark; ^4^ Department of Plant PathologyNorth Carolina State UniversityRaleighNCUSA

**Keywords:** above‐ and belowground biomass, allocation, allometry, N:P ratio, nutrient balance, P limitation, Tibetan Plateau

## Abstract

In terrestrial ecosystems, atmospheric nitrogen (N) deposition has greatly increased N availability relative to other elements, particularly phosphorus (P). Alterations in the availability of N relative to P can affect plant growth rate and functional traits, as well as resource allocation to above‐ versus belowground biomass (*M*_A_ and *M*_B_). Biomass allocation among individual plants is broadly size‐dependent, and this can often be described as an allometric relationship between *M*_A_ and *M*_B_, as represented by the equation $M_{A}=\alpha M_{B}^{\beta }$, or log *M*_A_ = logα + βlog *M*_B_. Here, we investigated whether the scaling exponent or regression slope may be affected by the N:P supply ratio. We hypothesized that the regression slope between *M*_A_ and *M*_B_ should be steeper under a high N:P supply ratio due to P limitation, and shallower under a low N:P supply ratio due to N limitation. To test these hypotheses, we experimentally altered the levels of N, P, and the N:P supply ratio (from 1.7:1 to 135:1) provided to five alpine species representing two functional groups (grasses and composite forbs) under greenhouse conditions; we then measured the effects of these treatments on plant morphology and tissue content (SLA, leaf area, and leaf and root N/P concentrations) and on the scaling relationship between *M*_A_ and *M*_B_. Unbalanced N:P supply ratios generally negatively affected plant biomass, leaf area, and tissue nutrient concentration in both grasses and composite forbs. High N:P ratios increased tissue N:P ratios in both functional groups, but more in the two composite forbs than in the grasses. The positive regression slopes between log *M*_A_ and log *M*_B_ exhibited by plants raised under a N:P supply ratio of 135:1 were significantly steeper than those observed under the N:P ratio of 1.7:1 and 15:1. Synthesis: Plant biomass allocation is highly plastic in response to variation in the N:P supply ratio. Studies of resource allocation of individual plants should focus on the effects of nutrient ratios as well as the availability of individual elements. The two forb species were more sensitive than grasses to unbalanced N:P supplies. To evaluate the adaptive significance of this plasticity, the effects of unbalanced N:P supply ratio on individual lifetime fitness must be measured.

## 1 Introduction

In recent decades, atmospheric nitrogen (N) deposition has greatly increased the availability of N relative to other elements in terrestrial ecosystems (Bobbink et al., 2010). Increased N inputs can potentially decrease biodiversity (Hautier et al., 2014; Lu, Mo, Gilliam, Zhou, & Fang, 2010), alter plant community composition (Bai et al., 2010; Harpole & Tilman, 2007; Stevens, Dise, Mountford, & Gowing, 2004), and drive changes in ecosystem processes and function (Galloway, 2005). The effects of altered absolute nutrient availability on ecosystem structure and functioning have been well documented. In contrast, the effects on plant performance and function of changes in the nutrient balance (e.g., the ratio of available N and P in soil) that may result from nitrogen deposition have drawn much less attention (Cardinale, Hillebrand, Harpole, Gross, & Ptacnik, 2009; Schindler, 2003; Sterner, Forman, Hendrixson, Hood, & Zimmer, 2002). Recent investigations have found that increases in N deposition due to the burning of fossil fuels and the addition of fertilizer to agricultural ecosystems have not been paralleled by a similar increase in phosphorus (P) inputs (Peñuelas, Sardans, Rivas‐Ubach, & Janssens, 2012; Vitousek, Porder, Houlton, & Chadwick, 2010). Thus, N limitation of plant growth may shift to P limitation as the N supply increases, as reported in several temperate and tropical ecosystems (Bobbink et al., 2010; Peñuelas et al., 2013; Tao & Hunter, 2012).

According to optimal allocation theory, plants should allocate more resources to organs that capture the most limiting resource and less to organs that are involved in obtaining nonlimiting resources (Niklas & Enquist, 2002; Poorter & Nagel, 2000; Weiner, 2004). Thus, variation in nutrient availability (e.g., N, P) is expected to affect the pattern of biomass allocation in plants (Poorter & Nagel, 2000; Poorter et al., 2012). For example, plants growing in high‐nutrient soils should allocate proportionally more photosynthates to aboveground parts (assuming that water is not limiting), thereby increasing photosynthesis and the ability to compete for light, whereas those in low‐nutrient environments should allocate proportionally more photosynthates to roots in order to increase nutrient acquisition (Ericsson, 1995; Grime, 1988). While N input generally has positive effects on aboveground biomass (*M*_A_) (DiTommaso & Aarssen, 1989; Gough, Osenberg, Gross, & Collins, 2000), it is possible that the ratio of N to P is more important than N alone for determining above‐ versus belowground biomass (*M*_B_) allocation (Graham & Mendelssohn, 2016). Consistent with this conjecture, root allocation decreased and litter production increased with increasing N:P supply ratio in five wetland *Carex* species (Güeswell, 2005). Moreover, the N:P ratio had greater effects on plant biomass than the absolute supply of N and P, even though the absolute supply of N and P also strongly influenced plant biomass (Güeswell, 2005).

Because resources allocated to one function or organ are not available to others, variation in allocation patterns has important implications for plant adaptation and fitness across environments. For example, if soil nutrient limitation induces a reduction in *M*_A_, fruit and seed production may also decline. In addition, changes in *M*_A_ versus *M*_B_ allocation can influence carbon dynamics and storage in terrestrial ecosystems (Hui & Jackson, 2006; Niklas, 2005; Poorter & Nagel, 2000). Therefore, the analysis of allocation patterns and their responses to environmental conditions (particularly to soil nutrients) is one of the best tools available for investigating plants’ responses to current conditions and for forecasting their responses to future conditions (Weiner, 2004).

Given that biomass allocation in individual plants is broadly size‐dependent, it is important that studies of the factors that affect resource allocation control for plant size rather than simply report the ratios or proportions of biomass allocated to alternative functions (Müller, Schmid, & Weiner, 2000; Weiner, 2004). Many size‐dependent allocation patterns fit an exponential relationship, here $M_{A}=\alpha M_{B}^{\beta }$, or, alternatively, log *M*_A_ = logα + βlog *M*_B_, where α is the scaling constant, and β is the regression coefficient, respectively. This model is the most widely used to evaluate biomass allocation at different ecological scales and is often referred to as “the allometric equation.” If an increase in *M*_A_ is not directly proportional to an increase in *M*_B_, then the scaling exponent or the regression slope (β) will deviate from 1.0 (Niklas, 2004; Pearsall, 1927), resulting in an allometric rather than an isometric relationship between *M*_A_ and *M*_B_.

**Figure 1:**
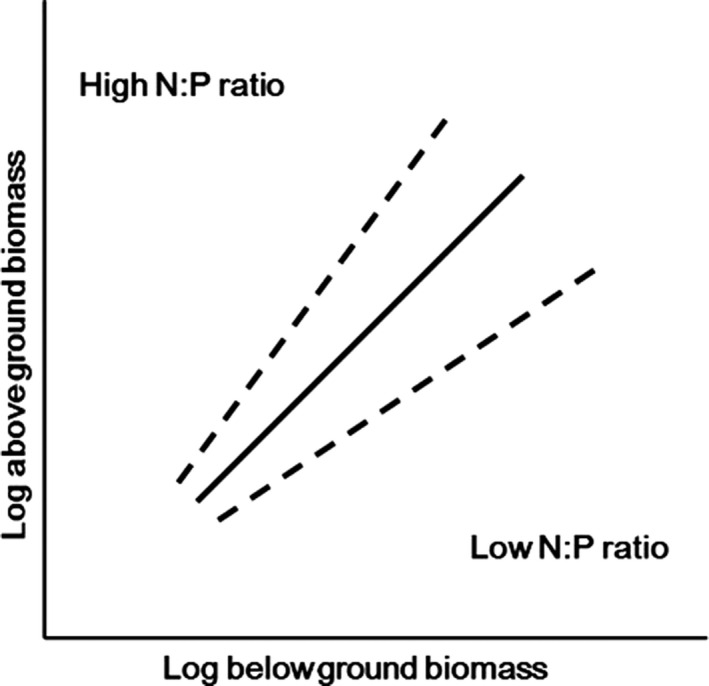
Hypothesized model: the exponent of the allometric function (i.e., the slope of log *M*_A_ = log α + βlog *M*_B_) should increase with the N:P supply ratio. This hypothesis was proposed because (1) root growth is usually more sensitive to P limitation than shoot growth (Güeswell, 2005; Venterink and Güsewell 2010); (2) plants exposed to the higher N:P supply ratios are expected to exhibit a bias toward elevated aboveground allocation; (3) under higher N:P supply ratio (P limitation), relatively larger plants would suffer P limitation more severely than smaller plants; under lower N:P supply ratio (N limitation), larger plants would suffer N limitation more severely than smaller plants. Accordingly, when P is deficient relative to N, root growth and development are expected to be more severely suppressed among larger individuals, contributing to an allometric relationship between *M*_A_ and *M*_B_

Allometric relationships both define and constrain how plants are constructed and how they function (Niklas, 2004). To date, ecologists have focused primarily on the independent effects of N or P inputs on variation in plant traits (e.g., *M*_A_ and *M*_B_), whereas relatively little attention has been paid to how the relative availability of N and P, expressed as the N:P supply ratio, affects the scaling relationship between *M*_A_ and *M*_B_. Based on a few previous studies that report that N or P limitation may affect plant belowground growth (Andrews, Sprent, Raven, & Eady, 1999; Carroll, Caporn, Johnson, Morecroft, & Lee, 2003; Güeswell, 2004; Müller et al., 2000; Yang, Fang, Ji, & Han, 2009), we hypothesize that the scaling exponent or slope of the relationship between *M*_A_ and *M*_B_ should change in response to a change in the N:P supply ratio, rather than the absolute level of N or P available. Specifically, we predict that plants experiencing a relatively high N:P supply ratio (and therefore P limitation) should allocate proportionally more resources to *M*_A_ with increasing plant size, thereby resulting in a larger scaling exponent and regression slope. Conversely, we predict that the scaling exponent and slope will be lower under conditions with a relatively low N:P supply ratio (Figure 1).

In this study, we selected five species that are common in alpine grasslands across the eastern Tibetan Plateau, three grasses and two composite forbs (Asteraceae), to test the allometric hypotheses proposed above. We conducted a greenhouse experiment to measure the effects of the N:P supply ratio on *M*_A_ and *M*_B_, and the scaling relationship between them. Forbs have been reported to decline in abundance in response to N addition (Clark et al., 2007; Gough et al., 2000; Harpole & Tilman, 2007). Possible reasons for the exclusion of composite forbs at high N are that grasses may tolerate a high N supply better than composites or that grasses outcompete composites at high N levels, but there is little experimental evidence to test these interpretations. In this study, we address the following questions:

1. How do above‐ versus belowground biomass allocation, specific leaf area (SLA), and tissue nutrient concentrations change in response to altered N and P supply ratios?
2. Does the scaling exponent between *M*_A_ and *M*_B_ increase with the N:P supply ratio?
3. Do species differ in their response to increases in N, P, and the N:P supply ratio with respect to *M*_A_ and *M*_B_? Do grasses perform better than forbs under conditions of either higher N availability or higher N:P supply ratios?

## 2 Material and Methods

### 2.1 Plant species and experimental conditions

**Figure 2:**
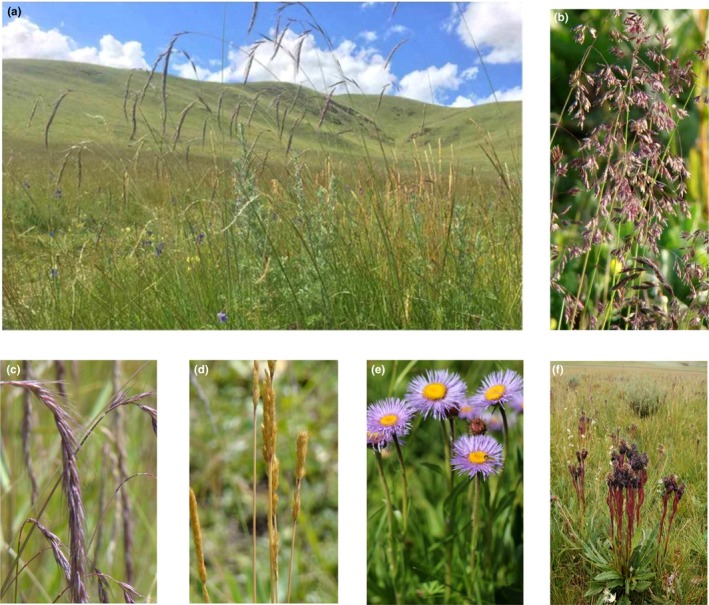
(a) The alpine meadow community in the eastern Tibetan Plateau; (b) *Poa crymophila* Keng.; (c) *Elymus nutans* Griseb.; (d) *Koeleria macrantha* (Ledeb.) Schult.; (e) *Aster diplostephioides* (DC.) C. B. Clarke; (f) *Saussurea nigrescens* Maxim

We selected three grasses (*Poa crymophila* Keng*, Koeleria macrantha* (Ledeb.) Schult*.,* and *Elymus nutans* Griseb.) and two composite forbs (*Aster diplostephioides* (DC.) C. B. Clarke and *Saussurea nigrescens* Maxim) that are broadly distributed in alpine meadows across the eastern Tibetan Plateau (35°58′N, 101°53′E; Figure 2). The seeds of each species were collected from two or three populations located 30–60 km apart in August 2014. More than 20 infructescences representing different maternal families from each population were sampled, placed in paper envelopes, and then stored at 4°C. After all of the seeds representing each species were thoroughly mixed, 90 seeds per species were selected and germinated in three petri dishes and kept moist with tap water under *c*. 50% daylight and 20°C in an incubator in spring 2015. After 2 weeks, 35 healthy seedlings per species were transplanted to 0.75‐l plastic pots filled with *c*. 300 g vermiculite and placed on flat trays in a greenhouse at Nanjing Agricultural University. The light provided in the greenhouse was 12‐hr day length and 900 Par, and the temperature was 25–30°C.The positions of the pots were randomized once every 2 weeks to control for spatial heterogeneity in light and temperature.

### 2.2 Nutrient treatments

Based on previous studies (Güeswell, 2005; Koerselman & Meuleman, 1996), the optimal N:P ratio for most plants is approximately 15:1 (i.e., N limitation occurs when N:P < 14; P limitation occurs when N:P > 16). We used this ratio as the Control treatment in the current experiment, and we created two high and two low N:P supply ratio treatments. The two high N:P treatments each represented a N:P supply ratio of 135:1; the first was created by increasing ninefold (relative to the Control) the amount of N supplied; this treatment is referred to below as the “135:1 (N addition)” treatment. The second high N:P treatment was created using one‐ninth the amount of P supplied relative to the Control; this treatment is referred to as the “135:1 (P reduction)” treatment. The two low N:P treatments each provided a N:P supply ratio of 1.7:1; the first was created using 1/9 the amount of N supplied in the Control treatment and is referred to as the “1.7:1 (N reduction)” treatment. The second low N:P treatment was created by increasing ninefold the amount of P supplied relative to the Control; this is referred to as the “1.7:1 (P addition)” treatment (Table 1). Thus, the experiment had a factorial design, with five species, five treatments, and seven replicates, resulting in a total of 175 individuals.

**Table 1:** The amounts of N and P supplied to individual plants in each experimental treatment. The N:P supply ratios of 1.7:1(P addition) and 135:1(P reduction) provided the same absolute amount of N as the Control (N:P supply ratio = 15:1); the N:P supply ratio of 1.7:1(N reduction) and 135:1(N addition) provided the same amount of P as the Control

N:P supply ratio	N (mg)	P (mg)
15:1	21.02	1.40
1.7:1(−N)	2.38	1.40
1.7:1(+P)	21.02	12.36
135:1(+N)	189.00	1.40
135:1(−P)	21.02	0.16

Beginning the first week of April 2015, 5 ml of nutrient solution was supplied to each pot once per week for 14 weeks. N was supplied as KNO_3_ and P as KH_2_PO_4_. All other essential elements were supplied at a constant concentration, using Hoagland's solution (Appendix S1). Plants were watered every 2 days, and each pot was leached with distilled water three times once every 3 weeks in order to prevent the accumulation of nutrients and toxic compounds.

### 2.3 Measurements

After the 14 weeks, all plants were harvested to determine total leaf area, *M*_A_ and *M*_B_, and the nutrient concentration of the above‐ and belowground tissues. Total leaf area of each individual plant was measured using a leaf area meter (LI‐3100C). Then, leaf area (mm^2^) was divided by leaf biomass (g) to calculate the specific leaf area (SLA). All harvested plant material was dried for 48 hr at 70°C prior to weighing.

The above‐ and belowground parts of each individual were analyzed separately to measure total N and P concentration. After the tissue was digested using a modified Kjeldahl procedure (1 hr at 200°C and 2 hr at 340°C) in a mixture of concentrated sulfuric acid, salicylic acid, copper, and selenium, concentrations of N and P were measured with a continuous‐flow analyzer (Auto Analyer AA3, GER; Güeswell, 2005).

### 2.4 Data analysis

The nested ANOVAs were conducted to detect the effects of the N:P supply ratio on plant traits, where *M*_A_, *M*_B_, leaf area, SLA, and above‐ and belowground nutrient concentrations were included as the dependent variables, and species or functional group, N:P supply ratio, and species (or functional group) * N:P supply ratio interaction were included as fixed independent factors. Across N:P supply ratios, the mean values of each trait were compared using Tukey's test.

Multivariate analyses were performed using general linear models to test how species, N:P supply ratio, and log *M*_B_ affect log *M*_A_. Two models were performed. In model 1, log *M*_A_ was the dependent variable, and N:P ratio, species, interactions between N:P supply ratio and species, and log *M*_B_ were included as the independent variables. Model 2 was similar, except that functional group was used instead of species as an independent variable. By comparing the adjusted *R* ^2^ values of the two models, we identified which model provided the better fit. All calculations were carried out using the statistical software JMP, version 7.0 (SAS Institute 2007).

Within each treatment, the allometric relationship between *M*_A_ and *M*_B_ was first analyzed by pooling the data from all individuals and species; the five species were then analyzed separately. We also examined the allometric relationship within each treatment. We used the log‐transformed version of the allometric equation:

$$M_{A}=\alpha M_{B}^{\beta }\to \log M_{A}=\log\alpha +\beta \log M_{B},$$

where the allometric exponent is the slope, and the log of the allometric coefficient is the y‐intercept of the linear function. Reduced major axis (RMA) regressions were performed to estimate allometric slopes and compare their differences among treatments (JMP, version 7.0).

## 3 Results

### 3.1 Effects of N and P supply on plant growth

Both functional group (grasses vs. composite forbs) and the N:P supply ratio significantly affected most plant traits (Table 2), but (with the exception of leaf N concentration) functional group consistently explained more variation in *M*_A_ and *M*_B_ than the N:P supply ratio (Table 2).

**Table 2:** Results of nested ANOVAs (*F* ratios and significance levels) conducted to detect the effects of the functional group (grasses vs. composite forbs) or species, N: P supply ratio, and their interaction on plant traits. Significance levels:***, *P <* .001; **, *P* < .01; *, *P* < .05; no symbol, *P *> .05

Source	*df*	*M*_A_ (g)	*M*_B_ (g)	Leaf area (mm^2^)	SLA (mm^2^/g)	Leaf N (mg/g)	Leaf P (mg/g)	Root N (mg/g)	Root P (mg/g)	Leaf N:P	Root N:P
Functional group	1	158.80***	128.19***	108.99***	20.74***	0.47	41.65***	53.07***	6.49*	30.04***	33.43***
Species [Functional group]	4	0.46	0.89	0.50	5.43**	1.36	3.77**	8.26***	1.80	4.26**	0.33
N:P supply ratio	4	10.60***	8.80***	10.77***	4.08**	4.05**	11.08***	5.23**	5.56**	11.61***	6.09**
adj. *R* ^2^		0.68	0.63	0.61	0.39	0.13	0.46	0.52	0.21	0.47	0.34

**Figure 3:**
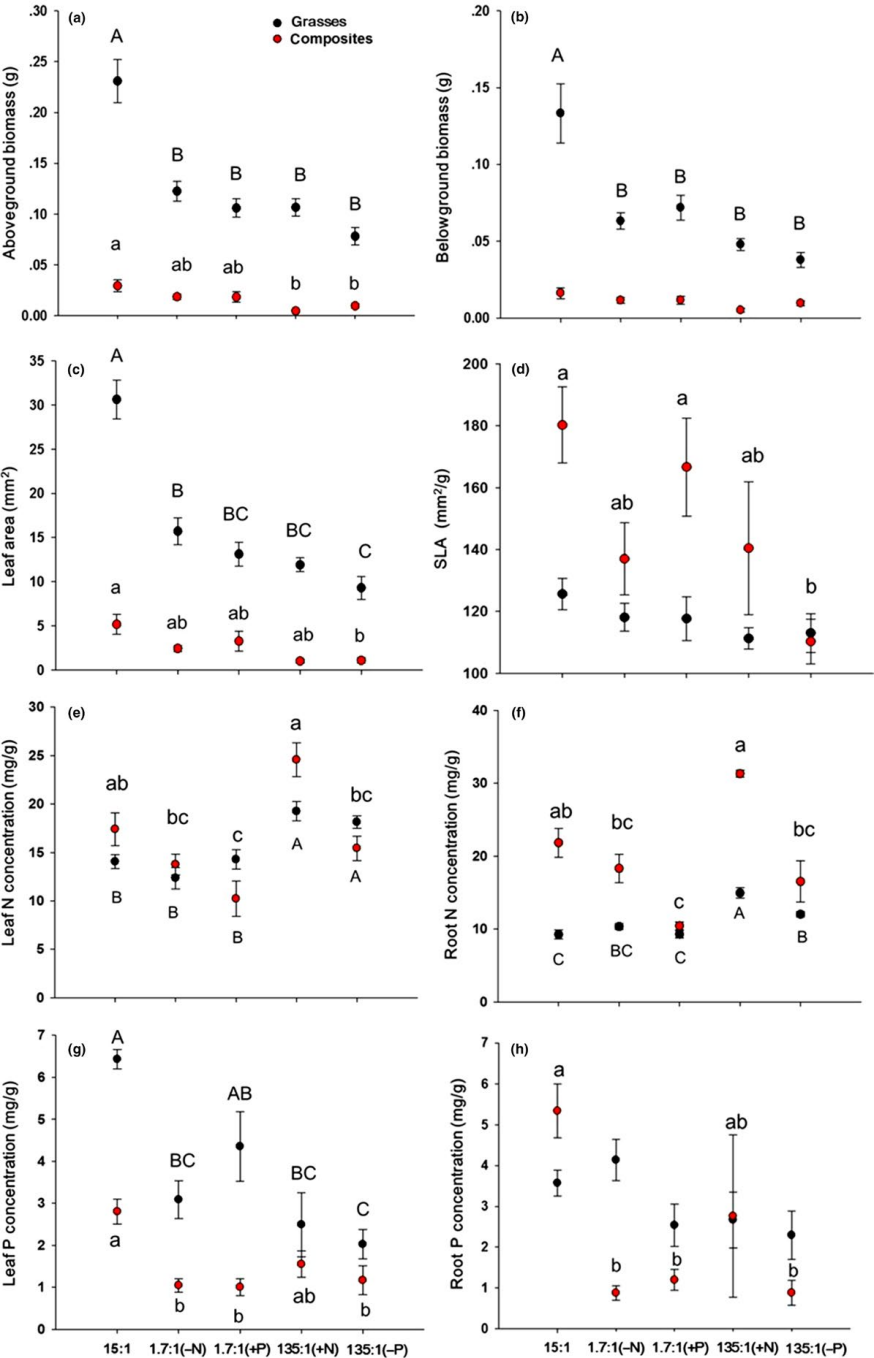
Mean trait values (± *SE*) of grasses (black symbols) and composite forbs (red symbols) measured in each experimental treatment after 14 weeks: (a) aboveground biomass; (b) belowground biomass; (c) leaf area; (d) specific leaf area (SLA); (e) leaf N concentration; (f) root N concentration; (g) leaf P concentration; and (h) root P concentration. The 15:1 treatment represents the balanced N:P ratio (the Control); the 1.7:1 (−N) treatment includes one‐ninth the amount of N as the Control; the 1.7:1 (+P) treatment includes nine times the amount of P as the Control; the 135:1(+N) treatment includes nine times the amount of N as the Control; the 135:1(−P) treatment includes one‐ninth the amount of P as the Control. Within each panel, means labeled with the same letter (capital letter: grasses; lower case: composites) do not differ at *P *=* *.05 according to Tukey's test. Letters were not shown when there was no significant difference between treatments

The unbalanced N:P treatments significantly reduced the mean *M*_A_ and *M*_B_ of the grasses (all individuals pooled) relative to the Control, but there were no significant differences among the unbalanced treatments (Figure 3a,b). Among the composite forb species (individuals pooled), the mean *M*_A_ values for the two 135:1 ratio treatments were significantly lower than the Control (Figure 3a), whereas the mean *M*_B_ did not differ significantly among treatments (Figure 3b). The *M*_A_ and *M*_B_ of the grasses greatly exceeded that of the forbs in all treatments, and the three grasses showed similar responses to the unbalanced N:P ratios, as did the two forbs (Appendices S2a,b; S3).

The mean total leaf area of grasses (individuals pooled) in each of the four unbalanced N:P treatments was significantly lower than that of the Control. The SLA of the grasses, however, did not differ significantly among treatments (Figure 3c,d). For the two composite forbs (individuals pooled), only the 135:1 (P reduction) treatment exhibited a significantly lower mean total leaf area and SLA than the Control (Figure 3c,d). When analyzing the five species separately, the total leaf area of *S. nigrescens* did not differ significantly among treatments (Appendices S2c and S3). In the three grasses, the SLA was not significantly affected by the N:P supply ratio, but in *A. diplostephioides*, the SLA was significantly lower in the 135:1 (P reduction) treatment than in the Control (Appendices S2d and S3). When all species were pooled, the effects of unbalanced N:P supply ratios on *M*_A_ and leaf area were similar to those in grasses (Figure 3a,c vs. Appendix S4).

### 3.2 Nutrient concentration

**Figure 4:**
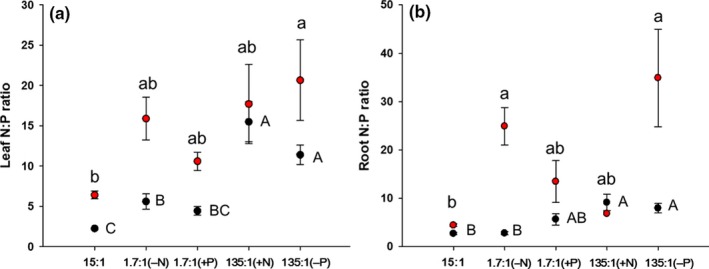
Mean values (± *SE*) of N:P in leaf (a) and root tissue (b) of grasses (black symbols) and composite forbs (red symbols) measured in each experimental treatment after 14 weeks. The 15:1 treatment represents the balanced N:P ratio (the Control); the 1.7:1 (−N) treatment includes one‐ninth the amount of N as the Control; the 1.7:1 (+P) treatment includes nine times the amount of P as the Control; the 135:1(+N) treatment includes nine times the amount of N as the Control; the 135:1(−P) treatment includes one‐ninth the amount of P as the Control. Within each panel, means labeled with the same letter (capital letter: grasses; lower case: composite forbs) do not differ at *P *=* *.05 according to Tukey's test

The N and P concentrations in leaf and root tissue differed significantly among nutrient treatments (Table 2). In the grasses, the 135:1(N addition) and 135:1(P reduction) treatments significantly increased leaf and root N concentration compared to the Control (Figure 3e,f). In the composite forbs, mean leaf and root N concentration were both significantly reduced by the 1.7:1(P addition) treatment, but they were not significantly affected by the other unbalanced N:P treatments (Figure 3e,f). The leaf P concentrations of grasses were significantly lower than the Control in the unbalanced N:P supply ratio treatments except for 1.7:1(P addition), but the root P concentration did not differ among treatments (Figure 3g,h). Relative to the Control, both the leaf and root P concentration of composites significantly declined under all unbalanced N:P treatments except for 135:1(N addition) (Figure 3g,h). In the grasses, only the 135:1 (both N addition and P reduction) treatments significantly increased the N:P ratios of leaf and root (Figure 4). In composites, the leaf N:P ratio was increased by the 135:1 (P reduction) treatment, whereas the root N:P ratio was increased in the 135:1 (P reduction) and 1.7:1 (N reduction) treatments (Figure 4).

The responses to the N:P supply ratio differed among species and traits. When analyzing the species separately, the 135:1 (N addition) treatment significantly increased root N concentration in all three grasses and in *A. diplostephioides*, and leaf N concentration in *K. macrantha* (Appendices S2e–h and S3); leaf P concentration was decreased in *P. crymophila* by the 1.7:1 (N reduction) treatment and in *E. nutans* and *A. diplostephioides* by the 1.7:1 (P addition), but increased in *K. macrantha* and *E. nutans* by 135:1 (P reduction) (Appendix S3). Root P concentrations were decreased by all the unbalanced N:P ratios in the two composites (except for 135:1 (N addition) in *S. nigrescens*), and by 135:1 ratios in *P. crymophila* and 1.7:1 (P reduction) treatment in *K. macrantha* and *E. nutans* (Appendix S3).

When all species were pooled, leaf N concentration was significantly increased by a higher N:P supply ratio, but only when the higher N:P ratio was achieved by adding N (Appendix S4). N concentration in the roots was significantly lower than the Control only in the 1.7:1 (P addition) treatment (Appendix S4). All of the unbalanced N:P treatments reduced leaf or root P concentration to a certain extent (Appendix S4). The N:P ratios of both leaf and root under the 135:1(both N addition and P reduction) supply ratios were significantly higher than the Control (Appendix S5).

### 3.3 Above‐ and belowground biomass allocation

The species or functional group, N:P supply ratio, their interaction (species or functional group*N:P ratio), and log *M*_B_ significantly affected log *M*_A_ (Table 3). Both models 1 and 2 accounted for most of the variation in log *M*_A_ and showed similar adjusted *R* ^2^, but the analysis based on species performed slightly better (Table 3).

**Table 3:** Multivariate analyses using general linear models. Model 1: dependent variable: log *M*_A_; factors: N:P supply ratio, species, interactions between N:P supply ratio and species, and log *M*_B_; Model 2: dependent variable: log *M*_A_; factors: N:P supply ratio, functional group, interactions between N:P supply ratio and functional species group, and log *M*_B_

	Source	*df*	F Ratio	Prob > *F*
Model 1 adj. *R* ^2^ = 0.9090	Species	4	26.33	<.0001
	N:P supply ratio	4	5.28	.0007
	Species*N:P supply ratio	16	3.34	.0001
	Log *M*_B_	1	129.07	<.0001
Model 2 adj. *R* ^2^ = 0.8752	Functional group	1	51.35	<.0001
	N:P supply ratio	4	7.34	<.0001
	Functional group*N:P supply ratio	4	3.71	.0069
	Log *M*_B_	1	97.62	<.0001

**Figure 5:**
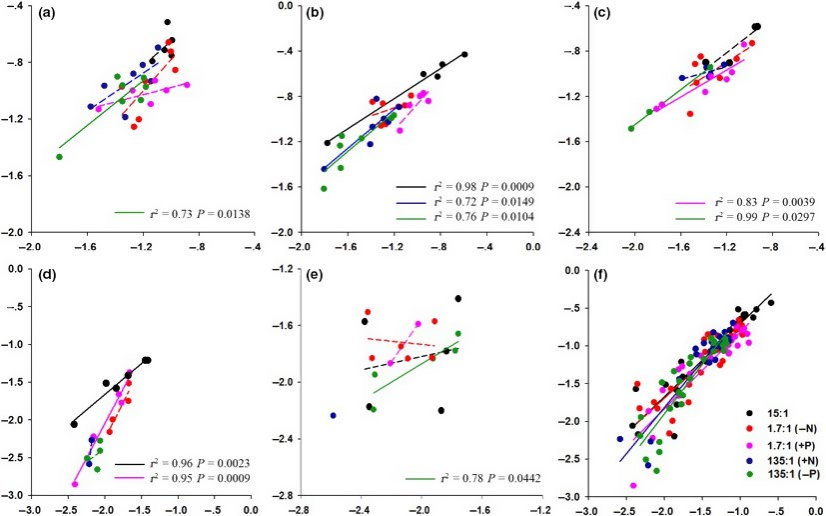
The linear regressions of log *M*_A_ versus log *M*_B_ in (a) *P. crymophila*, (b) *K. macrantha*, (c) *E. nutans*, (d) *A. diplostephioides* (e) *S. nigrescens,* and (f) across all species at different N:P supply ratios (solid lines represent regressions for which the slope is significantly >0; dashed lines represent nonsignificant relationships). The 15:1 treatment represents the balanced N:P ratio (the Control); the 1.7:1 (−N) treatment includes one‐ninth the amount of N as the Control; the 1.7:1 (+P) treatment includes nine times the amount of P as the Control; the 135:1(+N) treatment includes nine times the amount of N as the Control; the 135:1(−P) treatment includes one‐ninth the amount of P as the Control

When analyzing the five species separately, the slopes of the linear regressions between log *M*_A_ and log *M*_B_ in *P. crymophila*,* K. macranth*,* E. nutans,* and *S. nigrescens* were significantly greater than zero at 135:1(P reduction) (slope = 0.78, 0.86, 0.77, 0.64, respectively; Figure 5a,b,c, and e); the linear regressions in *K. macrantha* and *A. diplostephioides* were significant at 15:1 (slope = 0.66, 0.81; Figure 5b,d); the linear regressions in *E. nutans* and *A. diplostephioides* were significant at 1.7:1(P addition) (slope = 0.59, 1.83; Figure 5c,d); and the linear regression in *K. macrantha* was significant at 135:1(N addition) (slope = 0.86; Figure 5b). When all treatments were pooled, these regressions were significant in *P. crymophila* (slope = 0.71), *K. macrantha* (slope = 0.78), *E. nutans* (slope = 0.70), and *A. diplostephioides* (slope = 0.73). The slope of the regressions for *P. crymophila* and *E. nutans* was significantly <1.0, whereas the slope of the *A. diplostephioides* was significantly >1.0 (Appendix S6).

When all species were pooled, the relationships between log *M*_A_ and log *M*_B_ were significant and positive within all of the treatments (Figure 5f). The slopes of the regression lines at N:P ratios of 135:1 (N addition, slope = 1.22 and 135:1; P reduction, slope = 1.31) were significantly greater than the slope of the Control treatment (Table 4; Figure 5f). At N:P ratios of 1.7:1 (N addition) and 1.7:1 (P reduction), the slopes of the regression relationship between log *M*_A_ and log *M*_B_ were not statistically different from the Control (Table 4; Figure 5f).

**Table 4:** The scaling exponents (slope) and their respective 95% confidence intervals (CI) for the allometric model: log *M*_A_ = log α + βlog *M*_B_ at different N:P supply ratios for all species combined. The slope of the N:P supply ratio of 15:1 was treated as the Control (slope = 0.97) to test for significant variation among slopes representing altered N:P supply ratios. All slopes were significantly different from 0 (*P *< .0001). Slopes with asterisks are statistically significantly different from 0.97: **: *P *< .01; *: *P* < .05. Slopes in bold are statistically larger than 1.0 (*P *<* *.05)

N:P supply ratio	Sample size	*R* ^2^	Slope	Lower CI	Upper CI
15:1	31	0.86	0.97	0.887	1.211
1.7:1 (−N)	31	0.75	0.92	0.882	1.284
1.7:1 (+P)	25	0.83	1.01	0.931	1.324
135:1 (+N)	21	0.86	1.22*	1.107	1.561
135:1 (−P)	26	0.78	1.31**	1.217	1.789

Among the grasses (the three species pooled), the relationships were significantly positive in all of the treatments (Table 5). The 135:1 (P reduction) treatment exhibited a slope (0.74) that was significantly steeper than that of the Control (Table 5). Among the composites (two species pooled), the allometric relationship was significantly positive only in the Control and in the 1.7:1 (P reduction) treatment (Table 5).

**Table 5:** The scaling exponents (slope) for the allometric model: log *M*_A_ = log α + βlog *M*_B_ at different N:P supply ratios of two functional groups (grasses, composite forbs). Slopes with asterisks are statistically different from the Control (15:1): **, *P* < .001; *, *P *< .05

N:P supply ratio	Grasses				Composite forbs			
	Sample size	*R* ^2^	Slope	*P*	Sample size	*R* ^2^	Slope	*P*
15:1	20	.88	0.67	<.0001	11	.48	0.71	.01
1.7:1 (−N)	21	.37	0.63	.003	10	.00	0.03	.90
1.7:1 (+P)	18	.73	0.51	<.0001	7	.71	1.55	.01
135:1 (+N)	19	.58	0.73	.0001	3	.25	0.43	.66
135:1 (−P)	17	.78	0.74**	<.0001	9	.34	0.98	.09

## 4 Discussion

In the five species we examined here, experimental manipulation of the N:P supply ratio demonstrated that, for a variety of traits reflecting allocation of resources to above‐ and belowground organs, the N:P supply ratio had a stronger effect on mean phenotype than did the absolute supply of either N or P. In addition, the N:P supply ratio influenced the size‐dependent relationship between *M*_A_ and *M*_B_; across all species, when plants were exposed to the highest (135:1) N:P supply ratio, the allometric relationship between *M*_A_ and *M*_B_ became significantly more positive than that exhibited by plants exposed to an N:P supply ratio of 15:1 (the Control). Species‐specific analyses revealed considerable heterogeneity across species with respect to their plastic responses to variation in the N:P supply ratio and to levels of N and P (independent of the N:P supply ratio), and for many traits, the three grass species studied here exhibited much higher plasticity across treatments than did the two composite species.

### 4.1 Effects of N:P supply ratios on resource allocation to alternative functions

Neither N nor P addition alone [e.g., 135:1 (N addition) nor 1.7:1 (P addition)] increased *M*_A_ or *M*_B_ relative to the Control across all species (Figure 3a,b; Appendix S3). This result contrasts with previous findings that primary production is more strongly regulated by the availability of N than by P (de Groot, Marcelis, van den Boogaard, Kaiser, & Lambers, 2003). The decreased plant biomass induced by the unbalanced N:P supply ratios used here, however, is consistent with the hypothesis that P limitation could severely inhibit N uptake and further suppress plant growth. In the 1.7:1(P addition) treatment in grasses, plant growth may be also suppressed by the unbalanced N and P supply, even though the absolute N supply is the same as the Control (Figure 3a,b; Appendix S3). One possible interpretation is that excess P supply reduced root growth or performance, which subsequently inhibited N uptake. When pooling the species, equivalent N:P supply ratios had similar effects on plant traits (e.g., biomass and leaf area) independent of the absolute amounts of the individual elements supplied (Appendix S4).

Plants growing under high N:P supply ratios were expected to accumulate more starch, amino acids, or secondary compounds, and to increase leaf dry weight per unit area relative to plants growing in conditions of low N:P supply ratios (Atkinson, 1973; Vance, Uhde‐Stone, & Allan, 2003). With lower SLA, plants will capture less light per unit leaf weight and would be expected to exhibit weaker competitive ability (Funk, Standish, Stock, & Valladares, 2016). The reductions in SLA observed in forbs under the 135:1 (P reduction) treatment may reflect a trade‐off between resource investment in traits that can promote survival versus competition. In other words, under conditions characterized by an unbalanced elemental supply (particularly where P is limiting), a strategy of tolerance (rather than growth) may be a strategy for plants to survive. Testing this hypothesis, however, requires measures of the fitness consequences of variation in the SLA under a variety of N:P supply ratios. In the grasses, SLA was not similarly reduced by the altered N:P supply ratio, which suggests that grasses exhibited a stronger ability to capture light (and to maintain growth) than composite forbs under conditions with P deficiency.

N and P concentrations in plant tissue are determined by the balance between N and P uptake, C assimilation, and the losses of C, N, and P through turnover, leaching, exudation, herbivores, and parasites. In this study, the leaf N and root P concentrations of grasses differed little between equivalent N:P supply ratios characterized by different absolute levels of the N and P supply (Figure 3e,h). This supports the view that grasses have a homeostatic regulation mechanism that adjusts N and P uptake to meet the minimum requirement for growth (Yu et al., 2011). In the composite forbs, however, the leaf and root N concentrations differed significantly between the 135:1 (N addition) and 135:1 (P reduction) treatments (Figure 3e,g).

By comparing the N and P concentrations in living plant tissue, we found that plant P concentrations differed more among treatments than N concentrations. This result is consistent with Güeswell’s (2005) finding that variation in N:P ratios of root tissue was primarily determined by P concentration because N concentration is relatively stable (Güeswell, 2005).

### 4.2 Effects of the N:P supply ratio on the allometric relationship between *M*_A_ and *M*_B_

Resource allocation strategies in part reflect how plants adapt to their local environments and represent one way in which they may exhibit phenotypic plasticity in response to available nutrients (Müller et al., 2000). However, the biomass allocation patterns in plants may be less affected by changes in the absolute amount of nutrients supplied than by alterations in the N:P supply ratio. For example, previous greenhouse experiments and field investigations have found that the absolute nutrient supply had no significant effects on biomass allocation patterns (Enquist & Niklas, 2002; Müller et al., 2000; Yang et al., 2009).

As observed in the current study, the balance among essential elements can play an important role in controlling the biomass allocation pattern in plants. For example, at high N:P supply ratios (135:1), the slopes of the regressions of log *M*_A_ on log *M*_B_ were significantly steeper than among plants in the Control treatment, as we predicted (Figure 1). When exposed to unbalanced N:P supply ratios, and when all species were pooled, the relative growth rates of shoots and roots changed significantly, which may reflect a change in the nature of the trade‐off between allocation to *M*_A_ versus *M*_B_ (Weiner, 2004). To be specific, under high N:P supply ratios (135:1), the slopes of the linear regression between log *M*_A_ and log *M*_B_ increased significantly when all species were pooled.

Plants exposed to the higher N:P supply ratios exhibited a bias toward elevated aboveground allocation, suggesting that root growth was more sensitive to P limitation than shoot growth. In addition, the ability of plants to uptake nutrients may be primarily limited by P as plant size increases. When P was deficient relative to N, root growth and development were more severely suppressed among larger individuals, contributing to an allometric relationship between *M*_A_ and *M*_B_. It is possible that the effects of the N:P ratio reported here were mediated by changes in the soil microbial community. Although the soil microbial community (e.g., AMF) may change with nutrient availability and affect root growth (and, hence, belowground biomass), any such potential effects of soil microbial community on plant growth in the current study would have been indirect consequences of the change in nutrient supply.

The species pooled exhibited allometric relationships between *M*_A_ and *M*_B_ under all N:P supply ratios. The regression slope at 135:1 (P reduction) treatment was significantly steeper than the Control (Figure 5f; Table 4), which indicates that plants under P limitation (high N:P ratio) tend to allocate relatively less resources to roots with increasing plant size. Unfortunately, we could not compare the allometric relationships between grasses and composites with statistical rigor because the sample size for composites was very low due to their high mortality rate (Appendix S7).

In the current study, when N was deficient, larger plants did not allocate disproportionately more resources to roots, consistent with our prediction (Figure 1). Rather, they exhibited an allometric relationship between *M*_A_ and *M*_B_ (Table 4; Figure 5f). The responses of plant allocation to N versus P deficiency may have been mediated by cytokinins, which stimulate shoot growth. The effect of P supply on cytokinin concentrations is less rapid and pronounced than the effect of N supply, and cytokinin production is less influenced by available P when P is not limiting (de Groot et al., 2003). The responses of cytokinins to N versus P concentration in the rooting medium may be the mechanism by which relatively high N supply significantly reduced proportional root allocation, whereas high P supply did not (Figure 5f). In summary, the altered biomass allocation patterns observed in the current study appeared to result from P limitation (in the high N:P supply ratio treatment) induced by an unbalanced supply ratio.

The negative effects of elevated N input on species richness in communities with high productivity have been broadly studied (Aerts & Bobbink, 1999; Bobbink et al., 2010), but the role of P in affecting species persistence has received relatively little attention. In two studies, however, a larger number of endangered plant species were found under P‐ than under N‐limited conditions, which suggests that elevated P is more likely to be the cause of species loss than N enrichment (Fujita et al., 2014; Wassen, Venterink, Lapshina, & Tanneberger, 2005).

Our results suggest that plants respond to unbalanced nutrient supply ratios by adjusting the allometric relationship between *M*_A_ and *M*_B_. Although the relationships between *M*_A_ and *M*_B_ have been previously reported to be isometric and rarely affected by nutrient availability (Cheng et al., 2015; Niklas, 2004, 2005), the allometric pattern of biomass allocation found in the present study demonstrates that plants can be plastic in allocation and sensitive to altered N:P supply ratios.

Environment‐dependent allometric relationships between plant traits might be the product of fixed developmental responses of individuals within species rather than the result of natural selection, even if differences among species in such relationships are adaptive. While a better understanding of the physiological and evolutionary mechanisms that cause plants’ allometric allocation is needed, it is also clear that we need to pay more attention to the ratio among elements than to their absolute supply when studying plant life‐history strategy and their responses to global change.

### 4.3 Responses of grasses versus forbs to N, P, and N:P supply ratios: implications for plant communities

The change in available nitrogen can alter the interactions between species and cause shifts in plant dominance (Hillebrand, Bennett, & Cadotte, 2008; Suding et al., 2005). Usually, grasses become increasingly dominant under long‐term N addition due to their greater ability to take up nutrients relative to forbs (Chapin, Shaver, Giblin, Nadelhoffer, & Laundre, 1995; Mack, Schuur, Bret‐Harte, Shaver, & Chapin, 2004; Shaver et al., 2001). In our study, the three grasses exhibited significantly higher *M*_A_ and *M*_B_, and greater leaf area, than forbs under all unbalanced N:P supply ratios (Figure 3a–c). In addition, the two composite forbs had higher death rates than the grasses in response to higher N:P supply ratios, independent of the absolute amounts of supplied nutrients (Appendix S7). These results suggest that the composites examined here have a weaker ability than the grasses to survive and perform well when exposed to altered N:P supply ratios. Forbs would therefore be expected to become endangered or at greater risk of extinction than grasses when N:P supply ratios are unbalanced. Consistent with this, endangered species are often small and occur more frequently in environments characterized by high N:P ratios (Fujita et al., 2014).

Nutrient concentrations in plant tissue are often used to assess the availability of nutrients for plants and the degree to which particular nutrients are limiting (Mayor, Wright, & Turner, 2014). The results presented here are consistent with the interpretation that grasses are less sensitive to nutrient limitation induced by unbalanced N:P supply ratios than composite forbs (Figure 3e–h). With stronger homeostasis than composites, grasses may be more stable and more likely to become more dominant in grassland communities under altered N:P supply ratios. We have also conducted long‐term N and P addition treatments in alpine meadow ecosystems in the Tibetan Plateau to detect the stoichiometric responses of plants and found similar results (unpublished data). The N:P supply ratio should receive more attention in studies designed to detect the mechanisms of species coexistence and community assembly, and in studies that aim to forecast the species‐specific and community‐level consequences of N deposition.

## Conflict of Interest

None declared.
