# Supplementary material for: Nitrogen:phosphorous supply ratio and allometry in five alpine plant species
Source: Ecol Evol. 2016 Nov 22;6(24):8881–92. doi: 10.1002/ece3.2587 (PMC5192882; doi:10.1002/ece3.2587)

Appendix S1. The amount of essential elements other than N and P supplied to each plant.

| Other element | Amount  (mg pot−1 year−1) |
| --- | --- |
| K (KCl) | 332 |
| Ca (CaCl_2_) | 79.8 |
| Mg (MgSO_4_) | 25.6 |
| S (MgSO_4_) | 37.1 |
| Fe (Fe-EDTA) | 5.1 |
| Cu (CuSO_4_) | 0.01 |
| B (H_3_BO_3_) | 0.24 |
| Mn (MnCl_2_) | 0.55 |
| Mo (Na_2_MoO_4_) | 0.01 |
| Zn (ZnSO_4_) | 0.03 |

Appendix S2. Mean values (+ SE) of five species (*P. crymophila,* *K. macrantha,* *E. nutans,* *A. diplostephioides,* *S. nigrescens*) attributes measured in each experimental treatment after 14 weeks: (a) Above-ground biomass; (b) Below-ground biomass; (c) Leaf area; (d) Specific leaf area (SLA); (e) Leaf N concentration; (f) Root N concentration; (g) Leaf P concentration; and (h) Root P concentration. The 15:1 treatment represents the balanced N:P ratio (the Control); the 1.7:1 (-N) treatment includes 1/9 the amount of N as the Control; the 1.7:1 (+P) treatment includes nine times the amount of P as the Control; the 135:1(+N) treatment includes nine times the amount of N as the Control; the 135:1(-P) treatment includes 1/9 the amount of P as the Control.


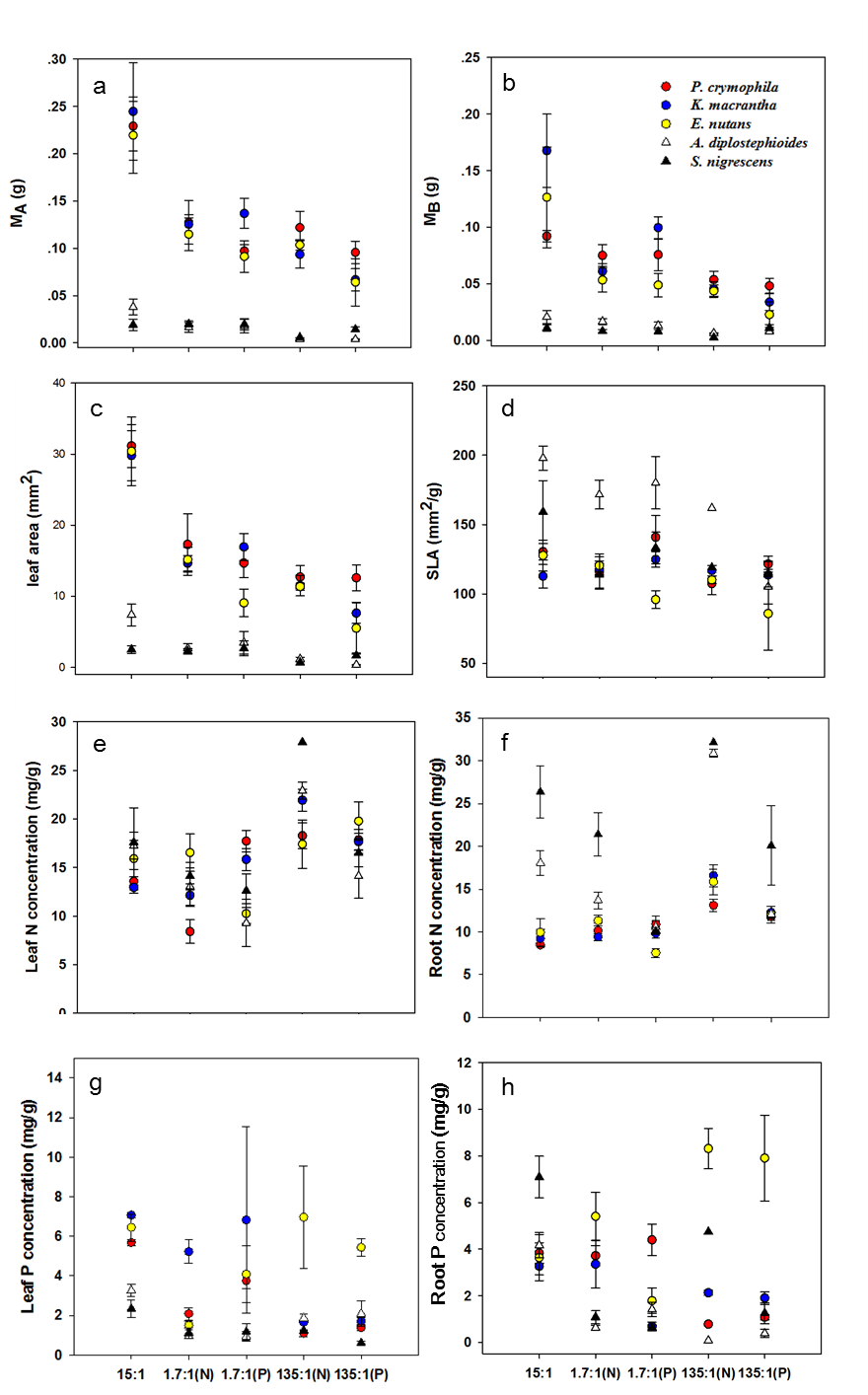


Appendix S3. Mean trait values of each species under different N:P supply ratio treatments. For each species and trait, means sharing the same superscript do not differ at *P* = 0.05 according to Tukey’s test.

|  | | Treatment | *P. crymophila* | | *K. macrantha* | | *E. nutans* | | *A. diplostephioides* | | *S. nigrescens* | |  |
| --- | --- | --- | --- | --- | --- | --- | --- | --- | --- | --- | --- | --- | --- |
| M_A_ (g) | | 15:1 | 0.2290 ^a^ | | 0.2446 ^a^ | | 0.2194 ^a^ | | 0.0378 ^a^ | | 0.0190 ^a^ | |  |
|  |  | 1.7:1(-N) | 0.1273 ^b^ | | 0.1253 ^b^ | | 0.1148 ^b^ | | 0.0163 ^ab^ | | 0.0200 ^a^ | |  |
|  |  | 1.7:1(+P) | 0.0971 ^b^ | | 0.1368 ^b^ | | 0.0912 ^b^ | | 0.0177 ^ab^ | | 0.0197 ^a^ | |  |
|  |  | 135:1(+N) | 0.1218 ^b^ | | 0.0934 ^b^ | | 0.1035 ^b^ | | 0.0040 ^ab^ | | 0.0058 ^a^ | |  |
|  |  | 135:1(-P) | 0.0955 ^b^ | | 0.0667 ^b^ | | 0.0641 ^b^ | | 0.0036 ^b^ | | 0.0142 ^a^ | |  |
| M_B_ (g) | | 15:1 | 0.0991 ^a^ | | 0.1676 ^a^ | | 0.1263 ^a^ | | 0.0205 ^a^ | | 0.0108 ^a^ | |  |
|  |  | 1.7:1(-N) | 0.0749 ^ab^ | | 0.0610 ^b^ | | 0.0532 ^ab^ | | 0.0166 ^a^ | | 0.0080 ^a^ | |  |
|  |  | 1.7:1(+P) | 0.0756 ^ab^ | | 0.0995 ^ab^ | | 0.0488 ^ab^ | | 0.0130 ^a^ | | 0.0078 ^a^ | |  |
|  |  | 135:1(+N) | 0.0535 ^ab^ | | 0.0452 ^b^ | | 0.0435 ^ab^ | | 0.0063 ^a^ | | 0.0026 ^a^ | |  |
|  |  | 135:1(-P) | 0.0481 ^b^ | | 0.0337 ^b^ | | 0.0227 ^b^ | | 0.0077 ^a^ | | 0.0111 ^a^ | |  |
| Leaf area (mm^2^) | | 15:1 | 31.15 ^a^ | | 29.80 ^a^ | | 30.39 ^a^ | | 7.38 ^a^ | | 2.49 ^a^ | |  |
|  |  | 1.7:1(-N) | 17.29 ^b^ | | 14.65 ^b^ | | 15.15 ^b^ | | 2.66 ^ab^ | | 2.27 ^a^ | |  |
|  |  | 1.7:1(+P) | 14.66 ^b^ | | 16.92 ^b^ | | 9.06 ^b^ | | 3.48 ^ab^ | | 2.69 ^a^ | |  |
|  |  | 135:1(+N) | 12.70 ^b^ | | 11.49 ^bc^ | | 11.35 ^b^ | | 1.15 ^ab^ | | 0.69 ^a^ | |  |
|  |  | 135:1(-P) | 12.59 ^b^ | | 7.62 ^c^ | | 5.51 ^b^ | | 0.35 ^b^ | | 1.66 ^a^ | |  |
| SLA (mm^2^/g) | 15:1 | | | 130.41 ^a^ | | 112.73 ^a^ | | 127.68 ^a^ | | 197.85 ^a^ | | 159.10 ^a^ | |
|  | 1.7:1(-N) | | | 116.11 ^a^ | | 117.77 ^a^ | | 120.38 ^a^ | | 171.60 ^a^ | | 113.95 ^a^ | |
|  | 1.7:1(+P) | | | 140.92 ^a^ | | 124.86 ^a^ | | 95.92 ^a^ | | 180.08 ^a^ | | 133.15 ^a^ | |
|  | 135:1(+N) | | | 107.42 ^a^ | | 116.78 ^a^ | | 110.10 ^a^ | | 161.90 ^ab^ | | 119.00 ^a^ | |
|  | 135:1(-P) | | | 121.48 ^a^ | | 113.58 ^a^ | | 85.80 ^a^ | | 105.20 ^b^ | | 114.32 ^a^ | |
| Leaf N  (mg/g) | 15:1 | | | 13.56 ^a^ | | 12.92 ^c^ | | 15.91 ^ab^ | | 17.25 ^a^ | | 17.58 ^a^ | |
|  | 1.7:1(-N) | | | 8.39 ^b^ | | 12..13 ^c^ | | 16.52 ^ab^ | | 13.04 ^ab^ | | 14.12 ^a^ | |
|  | 1.7:1(+P) | | | 17.70 ^a^ | | 15.83 ^bc^ | | 10.23 ^b^ | | 9.26 ^b^ | | 12.59 ^a^ | |
|  | 135:1(+N) | | | 18.27 ^a^ | | 21.92 ^a^ | | 17.40 ^ab^ | | 22.90 ^a^ | | 27.89 ^a^ | |
|  | 135:1(-P) | | | 17.84 ^a^ | | 17.66 ^b^ | | 19.77 ^a^ | | 14.11 ^ab^ | | 16.49 ^a^ | |
| Root N  (mg/g) | 15:1 | | | 8.46 ^c^ | | 9.23 ^b^ | | 9.94 ^b^ | | 18.06 ^b^ | | 26.36^a^ | |
|  | 1.7:1(-N) | | | 10.14 ^bc^ | | 9.39 ^b^ | | 11.30 ^b^ | | 13.68 ^bc^ | | 21.40 ^a^ | |
|  | 1.7:1(+P) | | | 10.88 ^abc^ | | 9.80 ^b^ | | 7.54 ^b^ | | 10.56 ^c^ | | 10.10 ^a^ | |
|  | 135:1(+N) | | | 13.10 ^a^ | | 16.58 ^a^ | | 15.85 ^a^ | | 30.87 ^a^ | | 32.14 ^a^ | |
|  | 135:1(-P) | | | 11.76 ^ab^ | | 12.27 ^b^ | | 12.01 ^ab^ | | 12.05 ^c^ | | 20.10 ^a^ | |
| Leaf P  (mg/g) | 15:1 | | | 13.56 ^a^ | | 12.92 ^c^ | | 15.91 ^ab^ | | 17.25 ^a^ | | 17.58 ^a^ | |
|  | 1.7:1(-N) | | | 8.39 ^b^ | | 12.13 ^c^ | | 16.52 ^ab^ | | 13.04 ^ab^ | | 14.12 ^a^ | |
|  | 1.7:1(+P) | | | 17.70 ^a^ | | 15.83 ^bc^ | | 10.23 ^b^ | | 9.26 ^b^ | | 12.59 ^a^ | |
|  | 135:1(+N) | | | 18.27 ^a^ | | 21.92 ^a^ | | 17.40 ^ab^ | | 22.90 ^a^ | | 27.89 ^a^ | |
|  | 135:1(-P) | | | 17.84 ^a^ | | 17.66 ^b^ | | 19.77 ^a^ | | 14.11 ^ab^ | | 16.49 ^a^ | |
| Root P  (mg/g) | 15:1 | | | 3.83 ^a^ | | 3.26 ^a^ | | 3.63 ^bc^ | | 4.16 ^a^ | | 7.09 ^a^ | |
|  | 1.7:1(-N) | | | 3.71 ^a^ | | 3.35 ^a^ | | 5.40 ^abc^ | | 0.62 ^b^ | | 1.08 ^b^ | |
|  | 1.7:1(+P) | | | 4.39 ^a^ | | 0.67 ^b^ | | 1.79 ^c^ | | 1.41 ^b^ | | 0.65 ^b^ | |
|  | 135:1(+N) | | | 0.78 ^b^ | | 2.12 ^ab^ | | 8.31 ^a^ | | 0.77 ^b^ | | 4.74 ^ab^ | |
|  | 135:1(-P) | | | 1.07 ^b^ | | 1.90 ^ab^ | | 7.90 ^ab^ | | 0.38 ^b^ | | 1.25 ^b^ | |

Appendix S4. Mean values (+ SE) of plant attributes (all species pooled) measured in each N:P ratio treatment after 14 weeks: (a) Above-ground biomass; (b) Below-ground biomass; (c) Leaf area; (d) Specific leaf area (SLA); (e) Leaf N concentration; (f) Root N concentration; (g) Leaf P concentration; and (h) Root P concentration. The 15:1 treatment represents the balanced N:P ratio (the Control); the 1.7:1 (-N) treatment includes 1/9 the amount of N as the Control; the 1.7:1 (+P) treatment includes nine times the amount of P as the Control; the 135:1(+N) treatment includes nine times the amount of N as the Control; the 135:1(-P) treatment includes 1/9 the amount of P as the Control. Within each panel, mean values labeled with the same letter do not differ at *P* = 0.05 according to Tukey’s test.


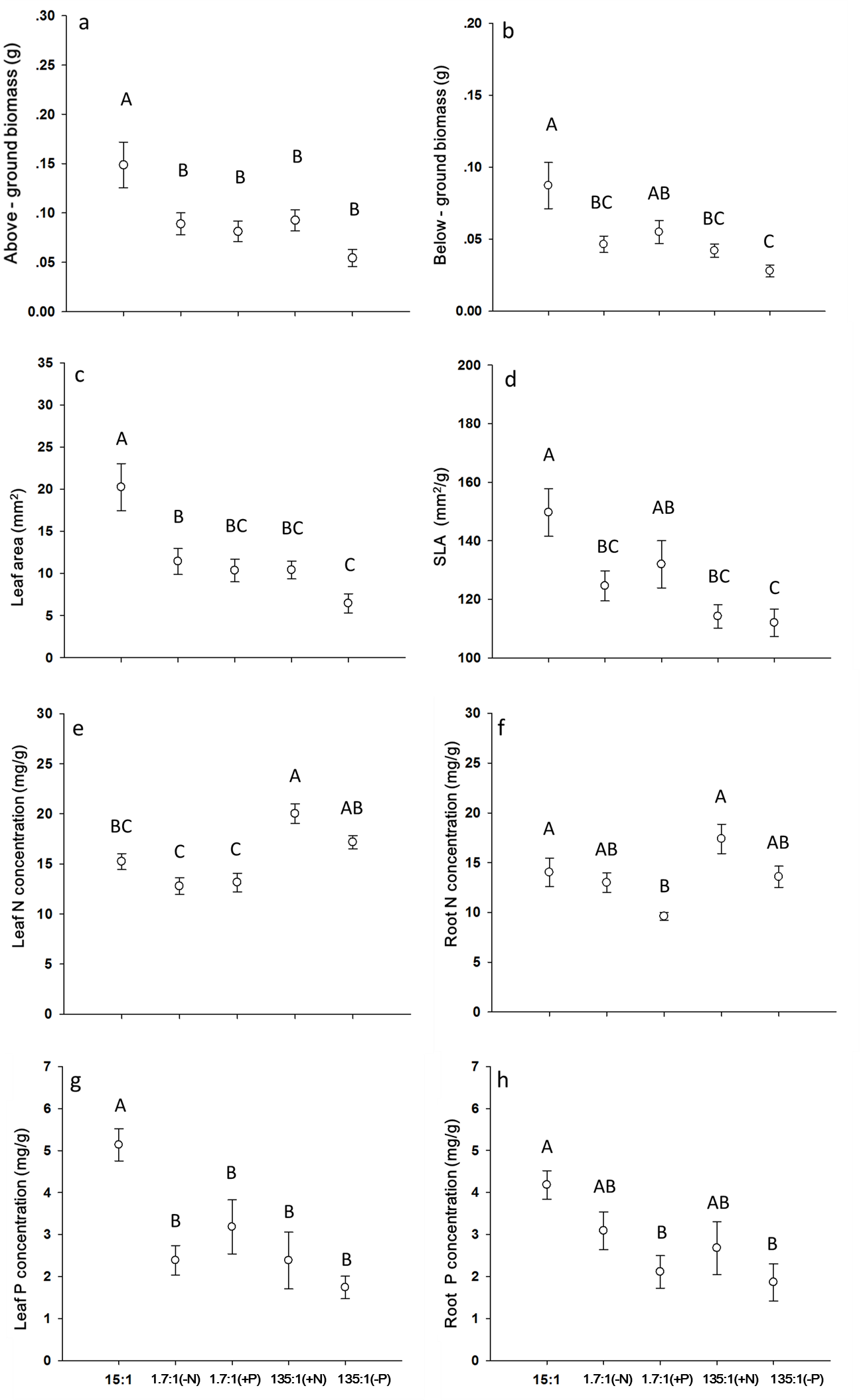


Appendix S5. Mean values (+ SE) of N:P in (a) leaf and (b) root tissue among all plants (species pooled) measured in each experimental treatment after 14 weeks. The 15:1 treatment represents the balanced N:P ratio (the Control); the 1.7:1 (-N) treatment includes 1/9 the amount of N as the Control; the 1.7:1 (+P) treatment includes nine times the amount of P as the Control; the 135:1(+N) treatment includes nine times the amount of N as the Control; the 135:1(-P) treatment includes 1/9 the amount of P as the Control. Within each panel, mean values labeled with the same letter do not differ at *P* = 0.05 according to Tukey’s test.


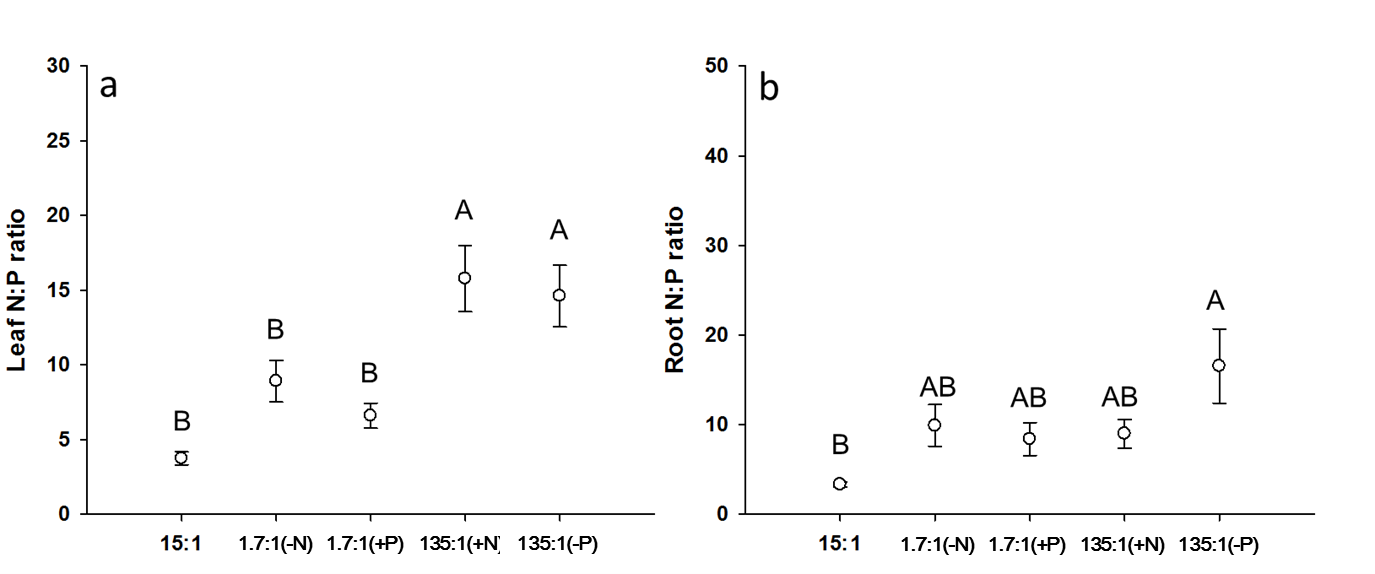


Appendix S6. The scaling exponents (slope) for the allometric model: log M_A_ = log α + βlog M_B_ of five species (all data pooled). Slopes with asterisks are statistically different from the 1.0; ^**^, *P* ＜ 0.001; ^*^, *P* ＜ 0.05.

|  | **Sample size** | **Slope** | **R^2^** | ***P*** |
| --- | --- | --- | --- | --- |
| *P. crymophila* | 32 | **0.71 (<1.0*)** | 0.49 | <0.0001 |
| *K. macrantha* | 33 | **0.78 (<1.0*)** | 0.82 | <0.0001 |
| *E. nutans* | 28 | 0.70 | 0.77 | <0.0001 |
| *A. diplostephioides* | 21 | **1.51 (>1.0***)** | 0.74 | <0.0001 |
| *S. nigrescens* | 19 | - | - | *-* |

Appendix S7. Mean mortality of grasses (black) and composites (red) measured in each N:P supply ratio treatment after 14 weeks. The 15:1 treatment represents the balanced N:P ratio (the Control); the 1.7:1 (-N) treatment includes 1/9 the amount of N as the Control; the 1.7:1 (+P) treatment includes nine times the amount of P as the Control; the 135:1(+N) treatment includes nine times the amount of N as the Control; the 135:1(-P) treatment includes 1/9 the amount of P as the Control.


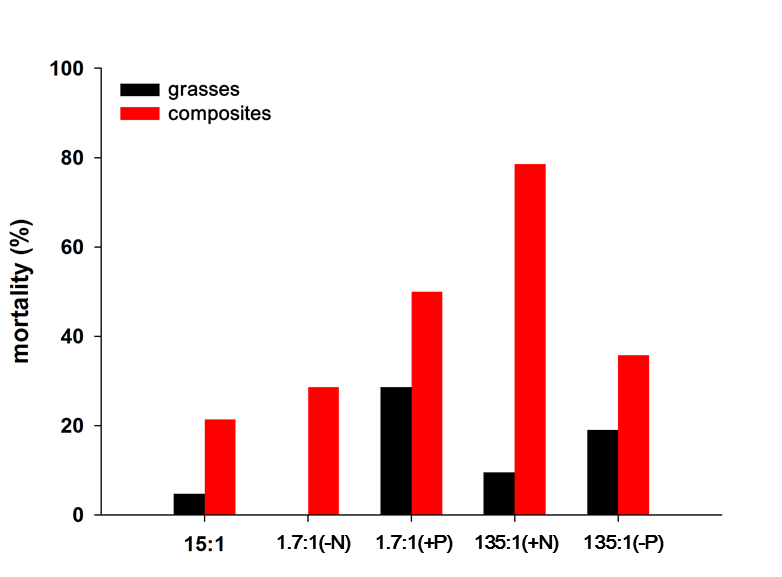

Supplement: Supplementary file 1 [file ECE3-6-8881-s001.docx]
